# Systematic screening of polyphosphate (poly P) levels in yeast mutant cells reveals strong interdependence with primary metabolism

**DOI:** 10.1186/gb-2006-7-11-r109

**Published:** 2006-11-15

**Authors:** Florian M Freimoser, Hans Caspar Hürlimann, Claude A Jakob, Thomas P Werner, Nikolaus Amrhein

**Affiliations:** 1 Institute of Plant Sciences, ETH Zurich, 8092 Zurich, Switzerland; 2 Institute of Microbiology, ETH Zurich, Zurich, Switzerland

## Abstract

**Background:**

Inorganic polyphosphate (poly P) occurs universally in all organisms from bacteria to man. It functions, for example, as a phosphate and energy store, and is involved in the activation and regulation of proteins. Despite its ubiquitous occurrence and important functions, it is unclear how poly P is synthesized or how poly P metabolism is regulated in higher eukaryotes. This work describes a systematic analysis of poly P levels in yeast knockout strains mutated in almost every non-essential gene.

**Results:**

After three consecutive screens, 255 genes (almost 4% of the yeast genome) were found to be involved in the maintenance of normal poly P content. Many of these genes encoded proteins functioning in the cytoplasm, the vacuole or in transport and transcription. Besides reduced poly P content, many strains also exhibited reduced total phosphate content, showed altered ATP and glycogen levels and were disturbed in the secretion of acid phosphatase.

**Conclusion:**

Cellular energy and phosphate homeostasis is suggested to result from the equilibrium between poly P, ATP and free phosphate within the cell. Poly P serves as a buffer for both ATP and free phosphate levels and is, therefore, the least essential and consequently most variable component in this network. However, strains with reduced poly P levels are not only affected in their ATP and phosphate content, but also in other components that depend on ATP or free phosphate content, such as glycogen or secreted phosphatase activity.

## Abstract 2

A systematic analysis of polyphosphate levels in yeast knockout strains for almost every non-essential gene identified 255 genes involved in the maintenance of normal polyphosphate content and provides insights into phosphate homeostasis.

## Background

Inorganic polyphosphate (poly P) is a linear polymer that consists of phosphoanhydride linked phosphate residues and occurs ubiquitously in all organisms and living cells [1]. The functions of poly P range from its role as a phosphate store and buffer [2-4] to the activation of enzymes [5,6] and regulation of chromatin condensation, gene expression and translation [1,7,8]. Poly P is also involved in bacterial pathogenicity [9,10], survival during stationary phase in bacteria and yeast [9,11,12], or the adaptation to alkaline and osmotic stress [13-15]. In the slime mold *Dictyostelium discoideum*, poly P regulates development and predation behavior [16], and in humans blood coagulation is accelerated and fibrinolysis is delayed by poly P [17]. At the cellular level, poly P has been detected in the cytosol, the nucleus, mitochondria, as well as the endoplasmic reticulum [7,18,19]. Poly P is particularly prominent in the acidocalcisomes of trypanosomatids [20], and in *Saccharomyces cerevisiae *almost the entire poly P pool resides in the vacuole [21,22].

Despite its universal occurrence and its broad functions, very little is known about poly P metabolism and its regulation. In *S. cerevisiae *(and all higher eukaryotes) it is, for example, still not known how poly P is synthesized, despite the fact that more than 20% of this organism's dry weight can be composed of poly P [1]. To gain a broad view of poly P metabolism in yeast and to identify pathways involved in the regulation of poly P levels, we extracted and quantified poly P in the knockout strains of almost all non-essential yeast genes.

## Results

Altogether, 4,765 strains from the YKO collection [23], consisting of knockouts of non-essential yeast genes, were initially screened. Strains that either hypo- or hyper-accumulated poly P were subjected to three consecutive rounds of screening. After the third round, 255 strains from the YKO collection had altered poly P levels in all three experiments (a complete list of all data is available online as Additional data file 1). Almost all of these 255 strains had reduced poly P levels and only occasionally, at specific time-points during growth, poly P hyper-accumulation relative to the wild-type strain was observed.

### All cellular compartments are involved in maintenance of poly P levels

**Figure 1:**
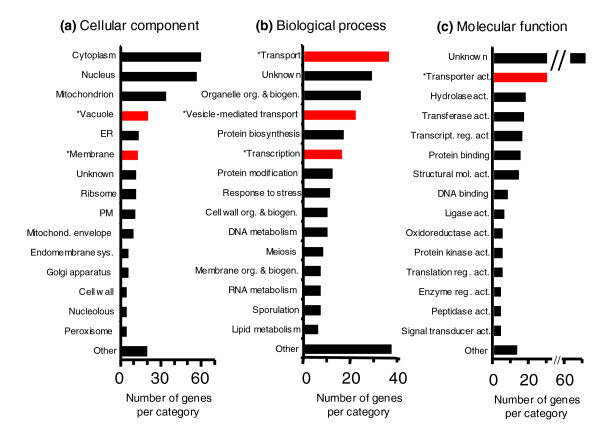
All cellular compartments are involved in the maintenance of poly P levels. Categorization of the 255 genes important for poly P levels by using the GO Slim terminology to: **(a) **cellular component, **(b) **a biological process and **(c) **a molecular function. Categories that were significantly overrepresented (P ≤ 0.05; red bars) are marked by an asterisk. Only the 15 most abundant categories are shown (all other genes are summarized as 'other').

All 255 discovered genes were categorized with the Gene Ontology (GO) Slim terminology and significantly overrepresented terms (relative to their occurrence in the whole yeast genome) were determined (Figure 1a-c). The cellular compartments with the highest number of proteins important for poly P content were the cytoplasm (59 proteins), the nucleus (56 proteins), the mitochondrion and the mitochondrial envelope (33 and 9 proteins, respectively), the vacuole (20 proteins) and the endoplasmic reticulum (13 proteins) (Figure 1a). Other genes found to be important for poly P content encoded membrane and ribosomal proteins (12 and 11 proteins, respectively) or proteins functioning in the Golgi apparatus (5 proteins), the peroxisome or the cell wall (4 proteins each) (Figure 1a). But only the terms 'vacuole' and 'membrane' were significantly overrepresented in the 255 genes that were found to be important for the maintenance of poly P levels. The representation of cellular compartments was also reflected in the highly overrepresented biological and molecular function terms: transport and vesicle mediated transport (36 and 22 proteins, respectively), transcription (16 proteins) and cell homeostasis (2 proteins) for biological function terms; and transporter activity (30 proteins) as the only significantly overrepresented molecular function term (Figure 1b,c). Other proteins encoded by the identified genes participate in biological processes such as organelle and cell wall organization and biogenesis (24 and 10 proteins, respectively), protein biosynthesis and modification (17 and 12 proteins, respectively), DNA and RNA metabolism (10 and 7 proteins, respectively) or are involved in the response to stress (11 proteins). Additional molecular functions that were prevalent among the discovered proteins included activities of hydrolases, transferases, transcription regulators and structural molecules (18, 17, 16 and 14 proteins, respectively) or protein and DNA binding proteins (15 and 8 proteins, respectively) (Figure 1c).

### Vacuolar proteins are most important in determining poly P levels

All 255 genes were ranked according to their impact on poly P content. The mutant cells most strongly affected in their poly P content were highly enriched for knockouts of genes that encode vacuolar proteins (6 out of the 20 vacuolar proteins among the 10 most strongly affected mutants). Vacuolar function depends on the acidification of the vacuolar lumen, which is mediated by the vacuolar H^+^-ATPase (V-ATPase) complex. In this poly P screen, 9 out of the 14 V-ATPase subunits were required for normal poly P content (Vma5 (14), Vma8 (10), Vma10 (13), Vma13 (34), Vph1 (1), Cup5 (5), Tfp3 (15), Ppa1 (20), Vma6 (27); ranks given in parentheses). In addition, regulators (Rav1 (43), Vps34 (30), Fab1 (150) and Vac14 (213)) and assembly factors (Vma22 (16), Vph2 (23)) of the V-ATPase were also discovered as being important for normal poly P levels.

Next to the *VPH1 *knockout, the *VTC4 *and *VTC1 *deletion strains were the second and third most affected mutants, and the knockout of *VTC2 *also hypo-accumulated poly P (rank 219). Together with Vtc3, these are the four subunits of the vacuolar transporter chaperon (Vtc) complex, which was previously shown to be required for maintenance of poly P levels [3]. Other proteins involved in the hypo-accumulation of poly P are involved in membrane docking and fusion at the Golgi-to-endosome and the endosome-to-vacuole steps (Vps33, ninth most affected mutant), or represent an alternative pathway from the Golgi to the vacuole (effected by the AP-3 complex that consists of Apm3, Apl5, Apl6 and Aps3; ranks 21, 59, 57 and 55, respectively). Vam3, a vacuolar t-SNARE protein that traffics to the vacuole via the AP-3 complex [24], was also important for maintenance of poly P levels (rank 8).

In other strongly affected strains, genes encoding either of the two phosphofructokinase subunits (*PFK1 *and *PFK2*, ranks 28 and 17) or the pyruvate kinase *PYK2 *(rank 7) were knocked out. In three additional strains, other components of glycolysis were identified (*GCR2*, *GPM3*, YOR283W; 63, 201 and 181, respectively).

### Poly P profiles of the identified mutant strains

**Figure 2:**
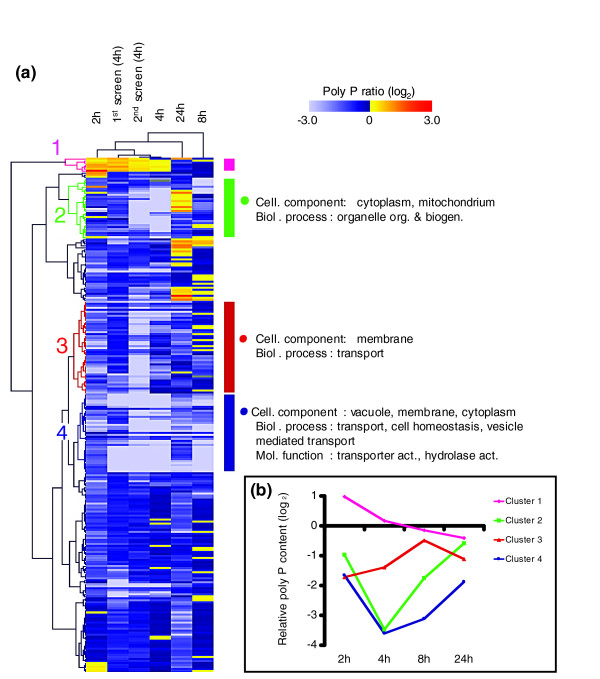
Clustering of all 255 genes found in this poly P screen resolved distinct groups with similar poly P profiles. **(a) **Hierarchical clustering (uncentered Pearson correlation, complete linkage) of all log_2_-transformed data (relative to the wild-type). Clusters significantly enriched (P ≤ 0.05) for any GO Slim category are marked by colored bars and branches. **(b) **Average poly P values (log_2_-transformed, relative to the wild type) for all genes in the four distinct clusters in (a) at the four time-points.

For the 255 discovered strains poly P data from six different experiments/time-points were available: the first and second screen (performed at the 4 h time-point) and the four samples from the third screen (2 h, 4 h, 8 h and 24 h samples). Hierarchical clustering of the six experiments with all log_2_-transformed poly P data revealed that the 4 h samples of the three experiments grouped together (Figure 2d). The 2 h time-point was more similar to the 4 h time-point than to the 8 h and 24 h samples (Figure 2). Clustering of the genes resolved distinct groups of genes with similar poly P patterns. One cluster of genes (Figure 2, cluster 1) comprised seven genes that caused elevated poly P levels at the 2 h and 4 h time-points when deleted. Three of the seven genes in this cluster (*KRE1*, *ECM33 *and *RIM21*) encode proteins that function in cell wall biosynthesis or organization, but no GO Slim category was significantly enriched in this cluster (Figure 2a). In two other clusters (Figure 2, clusters 2 and 4) the relative poly P content was, on average, minimal at the 4 h time-point and higher at 2 h and 24 h (Figure 2b). Cluster 2 was significantly enriched (compared to the complete genome) in cytoplasmic and mitochondrial proteins and in proteins functioning in organelle organization and biogenesis (Figure 2a). The mitochondrial proteins in cluster 2 comprised, for example, a mitochondrial phosphate transporter (Mir1) or three mitochondrial ribosomal proteins (Mrpl33, Mrp51 and Mrpl27). Cluster 4 contained the genes that caused the most dramatic effect on poly P levels when deleted: 22 genes in this cluster (out of a total of 39) were among the 30 most important genes for the maintenance of poly P levels. This group of genes also included six of the nine V-ATPase subunits (Vma8, Ppa1, Cup5, Vma10, Vph1, Vma5) that we discovered and many additional components of the vacuole. Correspondingly, this cluster was significantly enriched in vacuolar, membrane and cytoplasmic proteins that are involved in transport, cell homeostasis and vesicle mediated transport or exhibit transporter or hydrolase activity (Figure 2a). In the distinct cluster 3, membrane proteins that function in transport were also significantly overrepresented, but, on average, the relative poly P content varied only slightly or even increased during growth of the corresponding deletion strains (Figure 2b).

### Many strains with altered poly P levels are also affected in their total phosphate content, ATP levels, acid phosphatase activity and glycogen accumulation

Comparison of the 255 non-essential ORFs found to be important for poly P content with the results from published large scale analyses revealed that 56 strains were also affected in glycogen accumulation [25], but that only 4 strains with reduced poly P levels had previously been found in a screen for altered *PHO5 *(acid phosphatase (rAPase)) regulation, which serves as an indicator of the activation state of the *PHO *pathway [26,27]. To verify the data from the screen, and to test if a poly P phenotype is accompanied by other complex phenotypes, we measured cell density, poly P levels, total phosphate content, ATP concentrations, glycogen levels and rAPase activity in the 30 most affected knockout strains.

**Figure 3:**
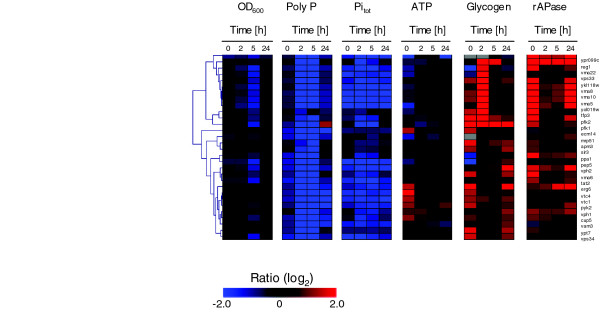
Many strains with altered poly P levels are also affected in their total phosphate content, ATP levels, rAPase activity and glycogen accumulation. All measurements (OD_600_, total phosphate content, ATP levels, rAPase activity and glycogen content) in the 30 strains most affected in their poly P levels are given relative to the wild type (log_2_-transformed). The data were hierarchically clustered (uncentered Pearson correlation, average linkage).

Overall, cell density and ATP content were the least affected characteristics, poly P and total phosphate content were generally reduced, and glycogen levels and rAPase activity were increased (Figure 3).

Cell density was slightly reduced (to roughly half the OD_600_) in most strains, but only at the 2 h and 5 h time-points (Figure 3). Poly P content and total phosphate levels were strongly reduced in all 30 non-essential mutants except in the knockout of *TFP3 *(only slightly reduced poly P and total phosphate levels) and the Δ*ecm14*, Δ*mrp51*,Δ*apm3 *and Δ*sir3 *strains had almost normal content of total phosphate (Figure 3). The ATP levels exhibited the highest variability at the 0 h time-point, with a cluster of knockouts having an increased content (knockouts of *ERG6*, *VTC4*, *VTC1*, *PYK2*, *VPH1*, *CUP5 *and *ECM14*). Strongly reduced ATP levels were only measured in the knockouts of the dubious open reading frame (ORF) YPR099c and the uncharacterized ORF YOL019w (Figure 3). Almost all of the 30 knockouts hyper-accumulated glycogen, either at the 2 h or the 0 h and 24 h time-points. An exception was again the mutation in the dubious ORF YPR099c, which had undetectable glycogen levels at the 0 h and 2 h time-points (which could be explained by the effect on *MRPL51*, which is encoded on the opposite strand). Most of the 30 knockout mutants also exhibited strongly increased rAPase activity at the 0 h and 24 h time-points. Exceptions were only the knockouts of *ECM14*, *VTC4*, *VTC1 *and *VAM3 *(Figure 3).

## Discussion

Previous to the screen described here, only very few yeast mutant cells had been analyzed with respect to their poly P content and, to our knowledge, this is the first time that a specific metabolite, that is, poly P, was isolated and quantified in an almost complete mutant cell collection. After three consecutive screens, 255 knockout mutant strains with altered poly P levels were identified. The genes affected in these mutant cells encode proteins from all intracellular compartments and components functioning in many processes of primary metabolism. This broad analysis thus defined novel biological functions for about 250 yeast genes and allowed, for the first time, a global view of the pathways and processes affecting poly P metabolism in yeast. But not only were many genes required for the maintenance of a normal poly P content, strains that hypo- or hyper-accumulated poly P had often a reduced total phosphate content, altered ATP and glycogen levels and an up-regulated rAPase secretion. From this analysis we conclude that poly P content is an extremely sensitive parameter that is highly intertwined with primary metabolism.

However, the number of knockout mutant cells with reduced poly P levels was probably even underestimated: Several of the few mutants previously identified to hypo-accumulate poly P were not found in this screen. For example, the knockouts of *VMA4*, *VTC3*, *ARG82 *or *KCS1 *were not discovered, as well as all knockouts of members of the *PHO *pathway (*PHO3*, *PHO4 *or, for example, *PHO84*), which have been shown to convey hypo-accumulation of poly P [3,26,28]. This is an indication of the stringency of the screening conditions and selection criteria and suggests that the true number of non-essential genes involved in poly P metabolism is even higher than the 255 genes reported here. The reliability of the high-throughput screening procedure was also confirmed by the fact that all 30 strains from the YKO collection that were individually tested indeed showed strongly reduced poly P levels.

Although poly P has been observed in and functions in different organelles [7,18,19,29], 90% to 99% of all poly P is localized in the vacuole [1,21,22]. Consequently, we measure almost exclusively vacuolar poly P and the proteins from other organelles that were found in this screen must affect vacuolar poly P levels. Poly P storage is thus a central function of the yeast vacuole and, thus, mutants affected in vacuolar functions or morphology are likely to be impaired in their capability to store polyP. Important vacuolar functions include the maturation and activation of different proteins and physiological functions in the storage of metabolites and in cell homeostasis [30-32] that all depend on the V-ATPase. This large multimeric, partly membrane-embedded complex is conserved from yeast to man and is also relevant in human diseases such as osteopetrosis and distal renal tubular acidosis [33,34]. In this screen, nine subunits of the V-ATPase and several of its regulators were identified. In addition, V-ATPase activity is also glucose regulated; in the presence of glucose, the V-ATPase is functional while the absence of glucose causes reversible dissociation of the V0 and V1 subunits and thus V-ATPase is inactivated [35]. This phenomenon could explain the decline of poly P levels as soon as the growth medium is depleted for glucose [36]. However, based on these data it is impossible to conclude whether V-ATPase activity regulates poly P levels directly or indirectly. As poly P levels in a *VMA4 *knockout strain could be slightly restored by growing the cells in acid buffered growth medium, Ogawa *et al*. [3] concluded that V-ATPase itself was not essential for poly P metabolism. Instead, these authors suggested that the Vtc complex (consisting of Vtc1, Vtc2, Vtc3 and Vtc4), which regulates vacuolar membrane fusion, morphology and function [37,38], is directly involved in the synthesis of poly P in yeast [3]. The fact that mutants affected in various stages of retrograde and forward vesicle trafficking, as well as in autophagy, indicate that an intact secretory pathway is required for normal poly P content and supports the hypothesis that the Vtc complex influences poly P levels indirectly [38]. This secondary effect on poly P metabolism can be caused by: the mislocalization or misregulation of vacuolar proteins that are important for poly P synthesis and storage; or the impairment of poly P synthesis and transport along the secretory pathway. Thus, many pathways could indirectly affect poly P content via their impact on the vacuole or secretory pathway and, thereby, link seemingly unrelated pathways to poly P metabolism. The reduced poly P content in mutants of any one of the four subunits of the AP-3 complex, which mediates an alternative pathway from the Golgi to the vacuole [39] and is also medically relevant for one type of Hermansky-Pudlak syndrome [40], is another such case of indirect effects; mutations in proteins that are targeted to the vacuole via the AP-3 complex or that contain a putative di-leucine signal for AP-3 targeting hypo-accumulated poly P (data not shown).

**Figure 4:**
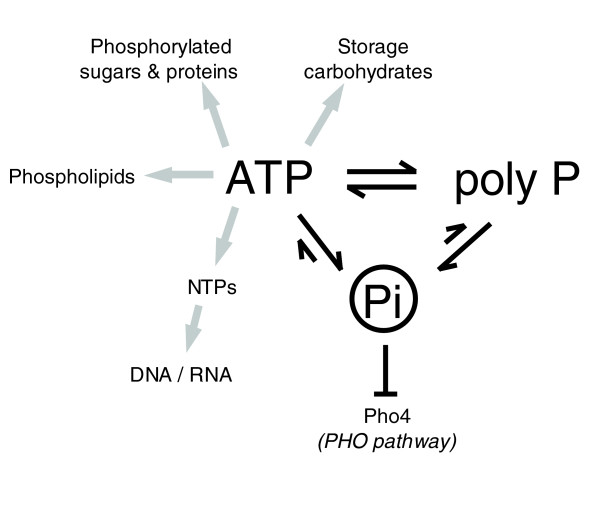
Poly P links energy and phosphate metabolism in a 'phosphate-energy-network'. Enzymatic reactions between the main phosphate/energy pools, ATP and poly P (shown by black arrows) are (at least partly) hypothesized. Flow to different organic phosphate/energy pools is indicated by the gray arrows. In contrast to ATP, poly P is less directly interconnected with the other phosphate pools and, therefore, its levels fluctuate more.

Although deletion of genes that encode vacuolar proteins caused the most severe reduction in poly P levels, only a fraction of all discovered genes encoded proteins that function in the vacuole. The remaining 235 genes encode proteins functioning in all cellular compartments. Assessing poly P metabolism within the larger picture of phosphate and energy homeostasis may help explain the involvement of this multitude of pathways. PolyP, which can constitute almost 50% of the total phosphate content in yeast (data not shown), is thus seen as another form of cellular phosphate besides free phosphate, DNA, RNA, nucleotides or phospholipids (Figure 4). Considering the example of poly P metabolism in *Escherichia coli *and the evidence for an ATP-dependent poly P kinase activity in yeast [7], it is assumed that the majority of yeast poly P is synthesized from ATP (Figure 4). The poly P pool is thus in direct equilibrium with the ATP pool, but can also be hydrolyzed to buffer free phosphate levels (Figure 4). ATP itself can also be hydrolyzed (for example, by the V-ATPase to assure vacuolar acidification). But more importantly, the ATP pool directly or indirectly sustains most cellular activities and processes such as DNA and RNA metabolism, ribosome biogenesis and assembly, transcription or protein biosynthesis, as well as synthesis of storage carbohydrates (Figure 4). Hence, these pathways are also indirectly associated with poly P metabolism. Ribosome biogenesis, for example, claims about 60% of total transcription, 90% of mRNA splicing, the requirement of all three RNA polymerases and the dedication of almost 200 different proteins and, thus, considerably affects energy homeostasis [41,42].

Intervention at any point within this phosphate-energy-network inevitably causes many side effects that require readjustment of the allocation of resources between the different phosphate and energy pools. In this context, 'interventions' could represent genetic changes but also externally induced physiological changes as they occur, for example, during the progression through different growth phases. Both types of changes were found to affect poly P levels strongly, but also other characteristics were altered. Many mutants that failed to accumulate poly P seemed to invest more in the accumulation of storage carbohydrates (glycogen) and could no longer buffer the expression of phosphate regulated genes (as indicated by elevated rAPase activity). In contrast, total phosphate content closely followed poly P levels, but even in this case the correlation coefficient between all relative poly P and all total phosphate values (log_2_-transformed) was only 0.65. Even ATP levels *per se *were sometimes significantly altered, but compared to the other characteristics that were measured, these changes were smaller, confirming, at least partly, the general notion that, in viable and growing cells, ATP levels remain constant [43].

## Conclusion

PolyP metabolism and primary metabolism are strongly interdependent: On one hand, poly P levels depend necessarily on the integrity of primary metabolism and reflect the physiological state of a cell. On the other hand, poly P itself influences cell metabolism through its importance for energy and phosphate homeostasis. This first genome-wide analysis of poly P content thus also implies poly P as an indirect link between different cellular pathways such as phosphate, energy and carbohydrate metabolism or transcription and translation activities.

## Materials and methods

### Strains and culturing conditions

The haploid yeast knock-out strains (YKO [23], based on the strain BY4741: MAT **a ***his3Δ1 leu2Δ0 met15Δ0 ura3Δ0*) were grown in 96-well deep well plates at 30°C in YPD medium (1% w/v yeast extract, 2% w/v peptone, 2% w/v glucose, with optional addition of 200 mg/l G418). For the screen, fresh YPD medium was inoculated with 40 μl of the precultured (3d) stationary cells and cells were harvested after 4 h (first and second round of screen) or after 2 h, 4 h, 8 h and 24 h (third round of screen). Growth and cell number was monitored by measuring the light scattering at 600 nm (OD_600_) in a BioTek PowerWave™ XS microplate spectrophotometer (BioTEK Instruments Inc., Vermont, USA). For detailed poly P measurements in selected strains, the culture was inoculated at a cell density of 1 OD_600_/ml (approximately 10^7 ^cells/ml), and at each time-point 1 OD_600 _equivalent of cells was collected for poly P quantification.

### Poly P purification and quantification

Poly P extraction, purification and quantification were performed as described previously [36]. In short, cells were pelleted in deep well plates by centrifugation (approximately 640 *g*, 15 minutes, 4°C), the supernatant was discarded, 50 μl 1 M sulphuric acid were added and the suspension was neutralized with 50 μl 2 M NaOH and 100 μl Tris-malate buffer (1 M, pH 7.5, 6% neutral red solution (0.1% neutral red in 70% ethanol)). Cell fragments were pelleted by centrifugation (approximately 640 *g*, 15 minutes, 4°C) and 100 μl of the supernatant were removed. Poly P was purified, enzymatically digested and quantified using a colorimetric assay [36].

### Screening procedure and data analysis

For all poly P measurements the poly P raw data were normalized to the cell density (OD_600_) measured for each corresponding culture. The poly P content was then expressed relative to either the median of all cultures in one 96-well plate (first screen) or the poly P content in the wild-type strain BY4741 (second and third screens). All strains with a relative poly P content 1.5-fold higher or a relative poly P content 0.67-fold lower were selected for the second and the third round of screening. In the third screen, data were collected at four different time points (2 h, 4 h, 8 h, 24 h). Only strains that fulfilled at least one of the following criteria were selected for subsequent data analysis: relative poly P content throughout the 24 h time-course varied more than two-fold; at least one time-point differed more than two-fold from the wild-type poly P content. Strains that did not grow were excluded. In some strains, the affected gene was reintroduced and expressed from a plasmid, which restored poly P levels (data not shown).

The poly P data of all selected strains and all screens and time-points were combined and clustered hierarchically (with the Pearson uncentered or Pearson correlation distance measure, respectively, and complete or average linkage in Genesis [44]). The selected strains were also ranked according to how strongly poly P levels were affected (calculated as the sum of the squares of the log_2_-transformed poly P ratios at the four time-points of the third screen).

The genes that were deleted in the strains with altered poly P content were categorized and studied by different computational tools and in comparison with available data sets. All genes were categorized according to the GO terminology by using the annotation given in the go_slim_mapping.tab file available from the *Saccharomyces *Genome Database [45]. Significantly overrepresented GOSlim terms (compared to their representation in the complete yeast genome) were determined by using Cytoscape and the BiNGO plugin (settings: hypergeometric test statistic, false discovery rate correction for multiple tests, P ≤ 0.05 confidence limit) [46,47]. All data manipulations were performed in Microsoft Excel and FileMaker.

### Measurement of total phosphate, ATP, glycogen and acid phosphatase activity

Detailed measurements of several metabolites and activities were performed in selected strains at various time-points (0 h, 2 h, 5 h, 24 h). Individual cultures for each time-point were inoculated to an OD_600 _= 1 (approximately 10^7 ^cells/ml). At each time point 1OD_600 _equivalent of cells was harvested and used for subsequent measurements. All data were obtained from duplicate measurements.

For measurement of total phosphate, cells were pelleted, resuspended in 200 μl of 1 MH_2_SO_4 _and heated in a boiling water bath for 20 minutes. Released phosphate was quantified with molybdate and malachite green as described [36]. For ATP quantification the same neutralized cell extract as for poly P quantification was prepared, and ATP was quantified by a method adapted from Hyswert *et al*. [48]. The neutralized sample (20 μl) were added to 80 μl Tris buffer (20 mM, pH8, 2 mM EDTA) and 4 μl phosphoenol pyruvate were added (2.5 mM, pH8, 0.125 M MgSO_4_, 0.312 M K_2_SO_4_). For the quantification of ATP, 5 μl of the sample were added to 45 μl luciferase buffer (10 mM Tris-H_2_SO_4_, pH 7.4, 3.5 mM MgSO_4_). After the addition of 50 μl luciferase solution (Roche ATP Bioluminescence Assay kit CLS II, Roche Diagnostics GmbH, Mannheim, Germany) relative light units emitted were measured in a luminometer (Lumat LB 9507, Berthold Technologies GmBH and Co. KG, Bad Wildbad, Germany).

To quantify glycogen the cell pellet was first frozen and glycogen was extracted as described [49]. The extracted glycogen was then digested by adding 10 U alpha-amylase and 12.6 U amyloglucosidase in a sodium acetate buffer (pH 4.8, 220 mM) at 55°C for 16 h. Released glucose was quantified with the D-Glucose HK kit (Megazyme International Ireland Ltd., Bray, Ireland).

Acid phosphatase activity was assayed according to Huang and O'Shea [27] in 50 μl cell suspension by adding 200 μl p-nitrophenyl-phosphate (20 mM). After 15 minutes at room temperature, 200 μl of 10% ice-cold trichloracetic acid and 400 μl sodium carbonate solution (2 M) were added and the OD_420 _was measured. All results were expressed relative to the respective values of the wild-type cells (BY4741).

## Additional data files

The following additional data are available with the online version of this paper. Additional data file 1 contains all poly P data of the three screens.

## Supplementary Material

Additional data file 1 All poly P data of the three screens. Click here for file
